# Tzeananiaceae, a new pleosporalean family associated with *Ophiocordycepsmacroacicularis* fruiting bodies in Taiwan

**DOI:** 10.3897/mycokeys.37.27265

**Published:** 2018-07-26

**Authors:** Hiran A. Ariyawansa, Alan J.L. Phillips, Wei-Yu Chuang, Ichen Tsai

**Affiliations:** 1 Department of Plant Pathology and Microbiology, College of Bio-Resources and Agriculture, National Taiwan University, Taiwan National Taiwan University Taipei Taiwan; 2 Universidade de Lisboa, Faculdade de Ciências, Biosystems and Integrative Sciences Institute (BioISI), Campo Grande, 1749-016 Lisbon, Portugal Universidade de Lisboa Lisbon Portugal

**Keywords:** Entomopathogenic fungi, Dothideomycetes, Multi-gene analysis, *Phoma*-like, Pleosporineae

## Abstract

The order Pleosporales comprises a miscellaneous group of fungi and is considered to be the largest order of the class Dothideomycetes. The circumscription of Pleosporales has undergone numerous changes in recent years due to the addition of large numbers of families reported from various habitats and with a large amount of morphological variation. Many asexual genera have been reported in Pleosporales and can be either hyphomycetes or coelomycetes. *Phoma*-like taxa are common and have been shown to be polyphyletic within the order and allied with several sexual genera. During the exploration of biodiversity of pleosporalean fungi in Taiwan, a fungal strain was isolated from mycelium growing on the fruiting body of an *Ophiocordyceps* species. Fruiting structures that developed on PDA were morphologically similar to *Phoma* and its relatives in having pycnidial conidiomata with hyaline conidia. The fungus is characterised by holoblastic, cylindrical, aseptate conidiogenous cells and cylindrical, hyaline, aseptate, guttulated, thin-walled conidia. Phylogenetic analysis based on six genes, ITS, LSU, *rpb2*, SSU, *tef1* and *tub2*, produced a phylogenetic tree with the newly generated sequences grouping in a distinct clade separate from all of the known families. Therefore, a new pleosporalean family Tzeananiaceae is established to accommodate the monotypic genus *Tzeanania* and the species *T.taiwanensis* in Pleosporales, Dothideomycetes. The *Ophiocordyceps* species was identified as *O.macroacicularis* and this is a new record in Taiwan.

## Introduction

We have been studying the families of Pleosporales considering both morphology and molecular phylogeny with the aim of providing a natural classification of this large order (Zhang et al. 2012, Hyde et al. 2013, Ariyawansa et al. 2013, 2014, 2015). *Phoma*-like asexual morphs have been shown to be scattered within the Pleosporineae, Pleosporales (Chen et al. 2017, Valenzuela-Lopez et al. 2018). While trying to resolve the natural classification of *Phoma*-like species in Pleosporales, several new families have been introduced within the sub-order Pleosporineae by various authors (Zhang et al. 2009, 2012, Hyde et al. 2013, Ariyawansa et al. 2015, Hernández-Restrepo et al. 2017, Valenzuela-Lopez et al. 2018).

The Pleosporales is considered to be the largest and the most diverse order of the class Dothideomycetes, comprising over 4700 species classified in 53 families (Hyde et al. 2013, Ariyawansa et al. 2015, Hernández-Restrepo et al. 2017, Valenzuela-Lopez et al. 2018). Pleosporalean species are characterised by pseudothecial ascomata usually with a papilla and a peridium composed of several layers of cells (Zhang et al. 2009, 2012, Hyde et al. 2013, Jaklitsch and Voglmayr 2016, Jaklitsch et al. 2017). Asci are bitunicate, usually fissitunicate and produced within a persistent hamathecium with or without pseudoparaphyses (Ariyawansa et al. 2013, 2014, 2015, Hyde et al. 2013). Ascospores are generally septate but vary in colour and shape, with or without a gelatinous sheath (Zhang et al. 2009, 2012, Hyde et al. 2013, Jaklitsch and Voglmayr 2016, Jaklitsch et al. 2017). Asexual morphs can be coelomycetous or hyphomycetous (Zhang et al. 2009, 2012, Hyde et al. 2013, Ariyawansa et al. 2014, 2015, Hernández-Restrepo et al. 2017, Valenzuela-Lopez et al. 2018). Members of Pleosporales are ubiquitous, occurring in various habitats and can be recognised as epiphytes, endophytes or parasites of living leaves or stems, hyperparasites on fungi or insects, lichenised or saprobes of dead plant stems, leaves or bark (Zhang et al. 2012, Hyde et al. 2013, Ariyawansa et al. 2014).

Pleosporales comprises the suborders Pleosporineae and Massarineae. (Zhang et al. 2009, 2012, Hyde et al. 2013). The suborder Massarineae was proposed by Zhang et al. (2009) and currently comprises 12 families (Tanaka et al. 2015). Pleosporineae contains numerous economically important plant and human pathogens and, at present, the suborder comprises 20 families (Valenzuela-Lopez et al. 2018).

Taiwan is an island located in the western Pacific Ocean and the importance of Taiwan’s rich diversity of fungal species has been often stated in Asian and global studies (Tsai et al. 2018). A number of studies have been conducted to elucidate the diversity of pleosporalean fungi associated with various hosts and habitats in Taiwan (Chang and Wang 2009, Yang et al. 2016, Tennakoon et al. 2018), but they have rarely investigated species of Pleosporales associated with entomogenous fungi. During our investigation of pleosporalean taxa in Taiwan, a *Phoma*-like fungus was isolated from mycelium growing on the fruiting body of an *Ophiocordyceps* species. The objective of the present study was to determine the taxonomic status of the isolated fungus and the *Ophiocordyceps* species, considering both morphological characters and DNA sequence data.

## Materials and methods

### Fungal isolation

During the course of an exploration of ascomycetous fungi in Nantou County, Taiwan (24°06'20"N, 121°11'13"E) in July 2017, fungal mycelium was observed developing on a fruiting body of an unidentified *Ophiocordyceps* species. The mycelium was transferred to and spread on a Petri-dish containing 2% water agar (WA) and incubated at 25 °C. Single conidial isolates were established from sporulating conidiomata in Petri-dishes containing WA. Germinated conidia were transferred separately to plates of PDA (Ariyawansa et al. 2016 a, b).

### Sample preparation and morphological observation

Morphological descriptions were made from isolates cultured on 2% potato dextrose agar (PDA; Difco). Preparations for microscopy were mounted in distilled water, observed with an Olympus BX51 microscope with differential interference contrast (DIC) illumination and at least 30 measurements per structure were noted. Voucher specimens were deposited in the herbarium of Department of Plant Pathology and Microbiology, National Taiwan University (NTUH). Living cultures are stored at the Department of Plant Pathology and Microbiology, National Taiwan University Culture Collection (NTUCC). Taxonomic descriptions and nomenclature details were deposited in MycoBank.

### DNA extraction, PCR amplification and sequencing

Single conidial isolates were grown on PDA for 28 days at 25 °C in the dark. Genomic DNA was extracted from the mycelium using the Bioman Fungus Genomic DNA Extraction Kit (Bioman) following the manufacturer’s protocol (BIOMAN SCIENTIFIC CO., LTD). For *Ophiocordyceps* species, single spore isolation was not successful. Therefore DNA was extracted directly from the ascomata using a DNA extraction kit (E.Z.N.A. Forensic DNA kit, D3591-01, Omega Bio-Tek) following the protocol of Ariyawansa et al. (2014).

PCR amplification was conducted in a 50 μl reaction volume containing 5–10 ng DNA, 0.8 units Taq polymerase, 1X PCR buffer, 0.2 mM d’NTP, 0.3 μM of each primer with the addition of 1.5 mM MgCl_2_ (Ariyawansa et al. 2014). The PCR reactions for amplification of the internal transcribed spacer regions 1 and 2 flanking the 5.8S nrRNA gene (ITS) (Schoch et al. 2012), were performed under standard conditions (White et al. 1990, Stielow et al. 2010). PCR conditions for amplification of the partial SSU (Small subunit of the nrRNA gene) and LSU (Large subunit of the nrRNA gene) followed the protocol of Ariyawansa et al. (2015). Amplification of partial β-tubulin (*tub2*), *rpb2* (partial RNA polymerase II second largest subunit gene) and *tef1* (partial translation elongation factor 1-α gene) followed the procedure of Woudenberg et al. (2013) and Ariyawansa et al. (2014). Primer sets used for these genes were as follows: ITS: ITS5/ITS4; LSU: LR0R/LR5; SSU: NS1/NS4; *tub2*: TUB4Rd/TUB4Fd (White et al. 1990, Liu et al. 1999, Sung et al. 2007) *tef1*: EF1-728F/EF1-986R (Carbone and Kohn 1999) and *rpb2*: fRPB2-SF/ fRPB2-7cR (Woudenberg et al. 2013). The PCR products were visualised on 1.5% agarose gels stained with SYBR-safe DNA gel stain. PCR products were purified and sequenced by Genomics, New Taipei, Taiwan. DNASTAR Lasergene SeqMan Pro v.8.1.3 was used to obtain consensus sequences from sequences produced from forward and reverse primers. Newly generated sequences were deposited at NCBI GenBank under the accession numbers provided in Suppl. material 1: Table 1.

**Table 1.** Comparison of alignment properties of genes and nucleotide substitution models used in Pleosporales phylogenetic analysis.

	LSU	SSU	*rpb2*	*tef1*	ITS	*tub2*
Alignment strategy (MAFFT v6)	G-INS-1	G-INS-1	G-INS-1 +manual	G-INS-1 +manual	G-INS-1 +manual	G-INS-1 +manual
Nucleotide substitution models for Bayesian analysis (determined by MrModeltest)	GTR+I+G	HKY+I+G	GTR+I+G	GTR+I+G	GTR+I+G	GTR+I+G

### Sequence alignment and phylogenetic analysis

Multiple sequence alignments were produced with MAFFT v. 6.864b (http://mafft.cbrc.jp/alignment/server/index.html). The alignments were checked visually and adjusted manually where required. Two different datasets were prepared to evaluate two phylogenies; a Pleosporales tree and a phylogeny of the genus *Ophiocordyceps*. The first tree focused on phylogenetic placement of the new family Tzeananiaceae introduced in this study in the Pleosporales and the second to determine the placement of the *Ophiocordyceps* species (NTUH 17-004) within the genus *Ophiocordyceps*. All introns and exons were aligned individually. Regions comprising various leading or trailing gaps were excluded from the ITS, LSU, *rpb2*, SSU, *tef1* and *tub2* alignments prior to tree building. All sequences obtained from GenBank and used by Hyde et al. (2013), Ariyawansa et al. (2015), Ban et al. (2015), Hernández-Restrepo et al. (2017), Wanasinghe et al. (2017), Valenzuela-Lopez et al. (2018) are listed in Suppl. material 1: Table 1. Single alignments for each locus and the combined six-gene dataset were analysed using different tree development methods.

Maximum parsimony (MP) analyses were made using PAUP v. 4.0b10 (Swofford 2002). Trees were inferred using the heuristic search option with 1000 random sequence additions. Maxtrees were unlimited, branches of zero length were collapsed and all multiple equally parsimonious trees were saved. Descriptive tree statistics for parsimony (Tree Length (TL), Consistency Index (CI), Retention Index (RI), Related Consistency Index (RC) and Homoplasy Index (HI)) were calculated.

Evolutionary models for each locus were determined individually using MrModeltest v. 2.3 (Nylander 2004) under the Akaike Information Criterion (AIC) implemented in both PAUP v. 4.0b10 and MrBayes v. 3.

A maximum likelihood analysis (ML) was executed at the CIPRES webportal (Miller et al. 2010) using RAxML-HPC2 on XSEDE (v 8.2.8) with default parameters and bootstrapping with 1000 replicates (Stamatakis 2014). The subsequent replicates were printed on to the best scoring tree obtained previously.

Bayesian Markov Chain Monte Carlo (MCMC) analyses were conducted in MrBayes 3.1.2 (Ronquist and Huelsenbeck 2003). The number of generations was set at 10 million and the run was stopped automatically when the average standard deviation of split frequencies fell below 0.01. Trees were saved each 100 generations. MCMC heated chain was set with a “temperature” value of 0.15. The distribution of log-likelihood scores was checked with Tracer v 1.5 to determine the stationary phase for each search and to decide if extra runs were required to achieve convergence (Rambaut and Drummond 2007, Ariyawansa et al. 2015). All sampled topologies below the asymptote (20%) were discarded as part of a burn-in procedure and the remaining trees were used to calculate posterior probabilities (BP) in the majority rule consensus tree.

**Figure 1.**
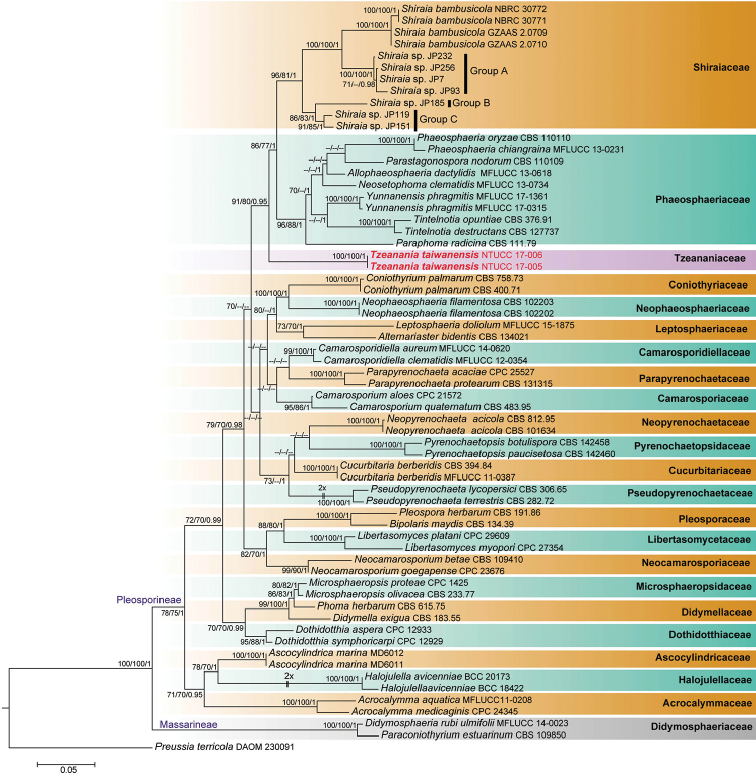
Phylogenetic tree (RAxML) obtained from the DNA sequence data of ITS, LSU, *rpb2*, SSU, *tef1* and *tub2* sequences of 64 strains showing taxa in suborders Massarineae and Pleosporineae within Pleosporales. The new isolates are shown in bold, red. MP and ML bootstrap values (BS) ≥70% and Bayesian posterior probabilities (PP) ≥0.95 are presented at the nodes. Several branches were shortened to facilitate presentation of the tree and this is indicated by two diagonal lines with the number of times a branch was shortened. The scale bar shows the number of estimated mutations per site. The tree was rooted to *Preussiaterricola* (DAOM 230091).

**Figure 2.**
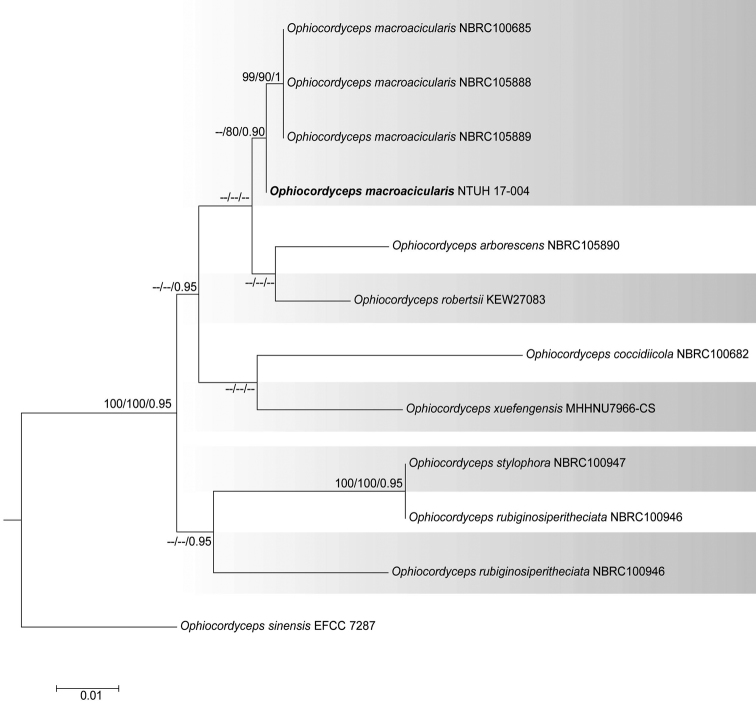
Phylogenetic tree (RAxML) obtained from the DNA sequence data of two loci (ITS and LSU) of *Ophiocordycepsmacroacicularis* and allied taxa. The new strain is shown in bold. MP and ML bootstrap values ≥70% and Bayesian posterior probabilities ≥0.95 are presented at the nodes and the scale bar shows the number of estimated mutations per site. The tree was rooted to *Ophiocordycepssinensis* (EFCC 7287).

Phylogenetic trees and data files were viewed in MEGA v. 5 (Tamura et al. 2011), TreeView v. 1.6.6 (Page 2001) and FigTree v. 1.4 (Rambaut and Drummond 2008). ML and MP bootstrap values equal to or greater than 70% and BP equal to or greater than 0.95 are given at each node in Figs 1, 2. Nodes with a posterior probability (PP) lower than 0.95 or MP and ML bootstrap support lower than 70% were considered unresolved.

**Table 2.** Comparison of alignment properties of genes and nucleotide substitution models used in *Ophiocordyceps* and allied species phylogenetic analysis.

	LSU	ITS
Alignment strategy (MAFFT v6)	G-INS-1	G-INS-1 +manual
Nucleotide substitution models for Bayesian analysis (determined by MrModeltest)	GTR+I	GTR+I+G

## Results

### Phylogeny

The data for the trees conducted in the different analyses are shown below. In the multi-gene analyses, the topologies of the trees acquired for the individual loci were checked visually to confirm that the overall tree topology of the single datasets were comparable to each other and to that of the tree obtained from the combined dataset alignment. Phylogenetic trees obtained from the combined gene analyses are supplied below (Figs 1, 2). Alignments were analysed corresponding to a single gene study of ITS, LSU, *rpb2*, SSU, *tef1* and *tub2* of the two phylogenies. Comparison of the alignment properties and nucleotide substitution models are provided in Tables 1, 2.

### Phylogeny of Pleosporales

The final alignment comprised 64 strains with 4558 characters (SSU 1019, LSU 877, ITS 450, *rpb2* 1013, *tef1* 902 and *tub2* 297). The maximum parsimony dataset consisted of 4558 characters of which 3226 were constant, 271 were variable and parsimony-uninformative and 1061 characters were parsimony-informative. Kishino-Hasegawa (KH) test showed length = 4234 steps, CI = 0.466, RI = 0.593, RC = 0.277 and HI = 0.534. The MCMC analysis of the six combined genes run for 66 × 10^4^ generations resulted in 6600 trees. The first 1320 trees, representing the burn-in phase of the analyses, were discarded, while the remaining trees were used to calculate posterior probabilities in the majority rule consensus tree.

A best scoring RAxML tree is presented in Fig. 1, with the Likelihood value of -20128.721105. Phylogenetic trees generated from ML, MP and Bayesian analyses produced trees with similar overall topology at subclass and family level relationships in agreement with earlier studies based on ML and Bayesian analysis (Hyde et al. 2013, Ariyawansa et al. 2015, Tanaka et al. 2015, Hernández-Restrepo et al. 2017, Wanasinghe et al. 2017, Valenzuela-Lopez et al. 2018).

The phylogenetic tree separated two distinct clades corresponding to the suborders Massarineae (represented only by the family Didymosphaeriaceae) and Pleosporineae (represented by more than 19 families). The two newly isolated strains from this study (NTUCC 17-005 and NTUCC 17-006) formed a distinct clade basal to the familial clades of *Shiraiaceae* and *Phaeosphaeriaceae* with high BS and PP support in analyses of the single locus and concatenated datasets. Hence, the novel lineage is regarded here as the new family Tzeananiaceae.

### Phylogeny of *Ophiocordyceps*

The final *Ophiocordyceps* alignment comprised 12 strains. The dataset consisted of 1523 characters (LSU 899 and ITS 624). The Bayesian analysis resulted in 1 × 10^4^ trees after 1 × 10^6^ generations. The first 2,000 trees, showing the burn-in phase of the analyses, were discarded, while the remaining trees were used to calculate posterior probabilities in the majority rule consensus tree.

The best scoring RAxML tree is shown in Fig. 2, with the Likelihood value of -3268.294101. Phylogenetic trees acquired from ML, MP and Bayesian analysis produced trees with similar overall topology at species level relationships in agreement with a former study based on ML and Bayesian analysis (Ban et al. 2015).

*Ophiocordycepsmacroacicularis* (NTUH 17-004), considered in this study, grouped in a well-supported clade with isolates NBRC 100685, NBRC 105888 and NBRC 105889 of *Ophiocordycepsmacroacicularis* that were used by Ban et al. (2015) to introduce the species, therefore confirming the identification of the studied species.

## Taxonomy

### Tzeananiaceae

Taxon classificationFungiPleosporalesTzeananiaceae

Ariyawansa, A.J.L. Phillips & Chuang fam. nov.

825566

#### Family description.

*Sexual morph*: undetermined. *Asexual morph*: *Conidiomata* pycnidial, solitary or aggregated, erumpent, globose, dark brown to black. *Conidiomatal wall* of *textura angularis*. *Conidiophores* reduced to conidiogenous cells. *Conidiogenous cells* phialidic, hyaline, smooth-walled, ampulliform. *Conidia* hyaline, cylindrical, guttulate.

### Tzeanania

Taxon classificationFungiPleosporalesTzeananiaceae

Ariyawansa, A.J.L. Phillips & Chuang gen. nov.

825567

#### Etymology.

Named after the Taiwanese mycologist, Shean-Shong Tzean, in recognition of his extensive contributions towards the taxonomy of entomopathogenic fungi.

#### Type species.

*Tzeananiataiwanensis* Ariyawansa, A.J.L. Phillips & Chuang.

#### Generic description.

*Sexual morph*: undermined. *Asexual morph*: *Conidiomata* pycnidial, partially or entirely immersed in the agar, solitary or aggregated, erumpent, globose. *Conidiomatal wall* of *textura angularis*. *Conidiophores* reduced to conidiogenous cells. *Conidiogenous cells* phialidic, hyaline, smooth-walled, ampulliform. *Conidia* hyaline, smooth- and thin-walled, cylindrical, guttulate. *Chlamydospores* not observed in culture.

### Tzeanania taiwanensis

Taxon classificationFungiPleosporalesTzeananiaceae

Ariyawansa, A.J.L. Phillips & Chuang sp. nov.

825568

Fig. 3

#### Type.

TAIWAN. Cueifong, Nantou County (24°06'20"N, 121°11'13"E), developing on a fruiting body of *Ophiocordycepsmacroacicularis*, 9 July 2017, Wei-Yu Chuang, (holotype: permanently preserved in a metabolically inactive state, NTUH 17-005!; culture ex-holotype NTUCC 17-005!).

#### Diagnosis.

Phylogeny based on ITS, LSU, *rpb2*, SSU, *tef1* and *tub2* revealed that the strains NTUCC 17-005 and NTUCC 17-006 considered in the present study formed a separate lineage sister to the familial clades of Shiraiaceae and Phaeosphaeriaceae in suborder Pleosporineae. Therefore, a new genus *Tzeanania*, a new species *T.taiwanensis* and a new family Tzeananiaceae in suborder Pleosporineae, Pleosporales are proposed here for the pycnidial coelomycete growing on the surface of the fruiting body of *Ophiocordycepsmacroacicularis*.

#### Etymology.

The epithet refers to Taiwan, where this species was collected

#### Description.

Developing on the fruiting body of *Ophiocordycepsmacroacicularis*.

*Sexual morph* not observed. *Asexual morph*: *Conidiomata* pycnidial, semi- or entirely immersed in the agar, solitary or aggregated, erumpent, globose, dark brown to black. *Conidiomatal wall* of *textura angularis*, 3–5 layered, composed of brown to dark brown, flattened polygonal cells. *Conidiophores* reduced to conidiogenous cells. *Conidiogenous cells* phialidic, hyaline, smooth-walled, ampulliform to globose, 3–5 × 0.5–2 μm, x¯ ± SD = 4 ± 0.7 × 1.5 ± 0.3 μm. *Conidia* hyaline, smooth-walled, thin-walled, cylindrical, guttulate, 4–6 × 1–2 μm, x¯ ± SD = 5.3 ± 0.27 × 1.5 ± 0.08 μm. *Chlamydospores* not observed in culture.

**Figure 3.**
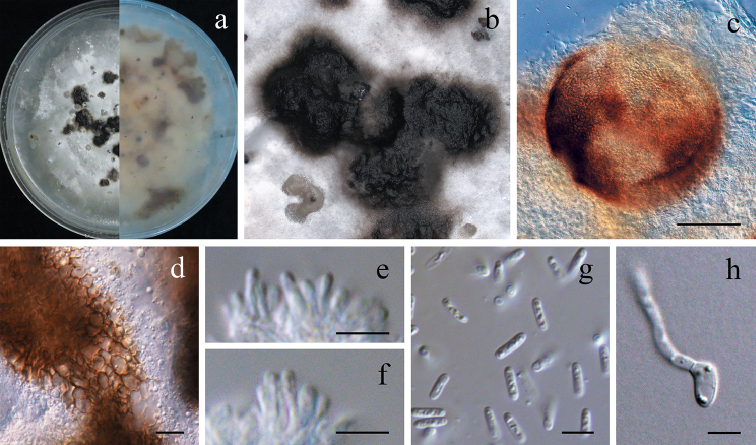
Morphology of *Tzeananiataiwanensis* (NTUCC 17-005) **a** Surface and lower view of colonies on PDA**b** Conidiomata sporulating on PDA**c** close-up of conidioma **d** close-up of Conidiomatal wall. **e–f** Conidiogenous cells **g** Conidia **h** Germinating conidia. Scale bars: 50µm (**c**), 10µm (**d**), 5µm (**e–h**).

#### Culture characteristics.

Colonies concentric circular pattern with radial furrows, entire, whitish, grey to olivaceous, with black conidiomata clustered in circular distribution; reverse concentric circular pattern with radial furrows, beige around centre and olivaceous at edge.

#### Distribution.

Taiwan

#### Additional material examined.

TAIWAN. Department of Plant Pathology and Microbiology, National Taiwan University, growing on a pine needles, 10 October 2017, Wei-Yu Chuang, (paratype: NTUH 17-006!, culture ex-paratype NTUCC 17-006!).

#### Notes.

*Tzeananiataiwanensis* differs from the familial type of Phaeosphaeriaceae, *Phaeosphaeriaoryzae* in having erumpent, globose conidiomata, conidiomatal wall 3–5 layered, with cylindrical, aseptate, hyaline conidiogenous cells and cylindrical, hyaline, aseptate, guttulated, thin-walled conidia. *Phaeosphaeriaoryzae* has immersed, uni- to multi-loculate, globose to subglobose conidiomata, conidiomatal walls comprising brown pseudoparenchymatous cells, with flattened ampulliform to doliiform, hyaline to pale brown conidiogenous cells and oblong to cylindrical, pale brown to brown, septate, smooth-walled guttulate conidia (Hyde et al. 2013).

Morphologically, *Tzeananiataiwanensis* differs from the familial type of Shiraiaceae, *Shiraiabambusicola* in having aseptate conidiogenous cells and cylindrical, hyaline, aseptate, guttulated, thin-walled conidia. *Shiraiabambusicola* has septate conidiogenous cells producing fusiform, muriform, hyaline to light brown, thick-walled conidia with irregularly arranged transverse and longitudinal septa (Hyde et al. 2013). Furthermore, *Tzeananiataiwanensis* can be clearly differentiated from *Shiraiabambusicola* by the host (*Ophiocordycepsmacroacicularis* versus Bamboo) and the distribution (Taiwan versus Japan and China).

## Discussion

In this study, a new family Tzeananiaceae is formally proposed in Pleosporineae, Pleosporales. This fungus was found on the surface of the fruiting bodies of *Ophiocordycepsmacroacicularis*. Phylogenetic analyses, based on DNA sequence data of ITS, LSU, *rpb2*, SSU, *tef1* and *tub2*, revealed it to form a separate lineage from all other families of Pleosporales. *Ophiocordycepsmacroacicularis* is reported for the first time from Taiwan. Moreover, our study expands the base of information regarding the diversity of pleosporalean fungi associated with entomogenous taxa in Taiwan.

Molecular data play a crucial part in present-day fungal systematics, but have some limitations (Ariyawansa et al. 2014, 2015, Hyde et al. 2014, Schoch et al. 2014, Thambugala et al. 2015). The most noteworthy and disconcerting question is that the phylogeny inferred from any one gene may not disclose the evolution history of the organism (Uilenberg et al. 2004). Taylor et al. (2000) proposed operational principles for Avise and Ball’s (1990) genealogical concordance species concept mainly for fungal taxa recognition. This Genealogical Concordance Phylogenetic Species Recognition (GCPSR) emphasised that species should be recognised based on genealogical concordance or genealogical non-discordance (Taylor et al. 2000, Dettman et al. 2003). This approach has been used to delineate species in several fungal groups (Udayanga et al. 2014, Manamgoda et al. 2014, Dettman et al. 2003, Ariyawansa et al. 2015). It is therefore better to integrate a polyphasic taxonomy with genotypic and phenotypic data in all forthcoming investigations (Uilenberg et al. 2004, Ariyawansa et al. 2014, 2015, Udayanga et al. 2014).

The family Shiraiaceae was introduced by Liu et al. (2013) to accommodate the bamboo parasitic genus *Shiraia* in suborder Pleosporineae. Phylogenetically, Shiraiaceae has close affinity with Phaeosphaeriaceae. Shiraiaceae species are mainly characterised by pinkish ascostromata that form on bamboo with many locules containing bitunicate asci each with six symmetrical, muriform ascospores (Hyde et al. 2013, Liu et al. 2013). The asexual morph is produced in immature ascostromata and form hyaline muriform, asymmetrical conidia (Hyde et al. 2013, Liu et al. 2013). *Shiraia* was introduced by Hennings (1900), based on *S.bambusicola*, as a monotypic genus. Later, Morakotkarn et al. (2008) reported several *Shiraia*-like strains, obtained from bamboo tissues as endophytes, which showed a close phylogenetic affinity to *Shiraiabambusicola*.

Phaeosphaeriaceae is one of the largest families in suborder Pleosporineae and includes economically important phytopathogens (Hyde et al. 2013). Species may also be found as endophytes or saprobes on different plant hosts, mainly on monocotyledons and several taxa have also been described on dicotyledons (Hyde et al. 2013). Members of Phaeosphaeriaceae are cosmopolitan and thus have been recorded from various regions around the world (Hyde et al. 2013).

Phylogenetically, *Tzeanania* has close affinity with Shiraiaceae and Phaeosphaeriaceae. To clarify the phylogeny of *Shiraia*-like fungal isolates, Morakotkarn et al. (2008) conducted a multi-gene phylogeny based on ITS, LSU and *tub2* and found three distinctive lineages, sister to *Shiraiabambusicola* clade, which were also identified with *Phoma*-like asexual morphs. Furthermore, Morakotkarn et al. (2008) concluded that *Shiraia*-like fungi Group A (Fig. 1) can be recognised as a novel species that could be allocated into a novel genus/species related to *S.bambusicola*. Single gene analysis of LSU and SSU showed that our strains formed a basal lineage to the familial clade of the Shiraiaceae. Therefore to confirm phylogenetic affinity of our isolates with *S.bambusicola* and *Shiraia*-like fungi groups A, B and C, we additionally conducted a comprehensive phylogeny derived from 3 genes LSU, ITS and TUB (data not shown). We produced a tree with similar topology to the one reported by Morakotkarn et al. (2008) while our new strains formed a distinct lineage sister to the familial clades of Shiraiaceae and Phaeosphaeriaceae, which further confirms the uniqueness of the new family Tzeananiaceae in suborder Pleosporineae.

**Figure 4.**
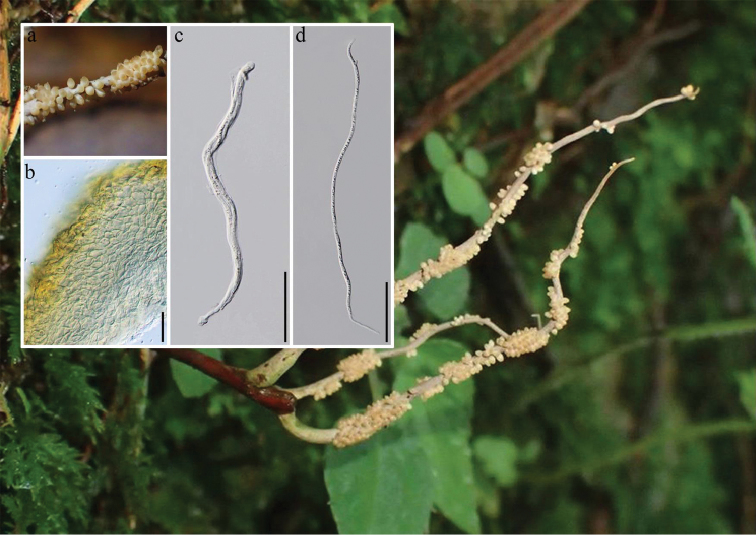
Morphology of *Ophiocordycepsmacroacicularis* (NTUH 17-004) **a** Close-up of ascomata **b** Close-up of the peridium **c** Hyaline, cylindrical, eight-spored ascus **d** Needle-shaped, multi-septate, hyaline ascospores. Scale bars: 20 μm (**b**), 50 μm (**c–d**).

*Ophiocordycepsmacroacicularis* S. Ban et al. was introduced by Ban et al. (2015) and was recently recorded from Thailand by Luangsa-ard et al. (2018) based on molecular phylogeny together with morphology (Figs 2, 4). To the best of our knowledge, this is the first record of *O.macroacicularis* in Taiwan. Sun et al. (2016) introduced a hyphomycetous taxon, *Calcarisporiumcordycipiticola*, which was also found to infect the fruiting bodies of *Cordycepsmilitaris* causing significant quality and yield losses. Even though we were able to obtain a single spore culture of *T.taiwanensis* (NTUCC 17-006) using the fruiting structures formed on PDA (Fig. 3b), single spore isolation of *O.macroacicularis* was not successful. Therefore, we could not clarify the exact nutritional mode of *T.taiwanensis* or its interaction with *O.macroacicularis*. Therefore, further studies are essential to understand the interaction between this unusual fungus and its host.

## Supplementary Material

XML Treatment for Tzeananiaceae

XML Treatment for Tzeanania

XML Treatment for Tzeanania taiwanensis

## References

[B1] Ariyawansa HA Jones EBG Suetrong S Alias SA Kang JC Hyde KD (2013) Halojulellaceae a new family of the order Pleosporales. Phytotaxa 130: 14–24. 10.11646/phytotaxa.130.1.2

[B2] Ariyawansa HA Tanaka K Thambugala KM Phookamsak R Tian Q Camporesi E Hongsanan S Monkai J Wanasinghe DN Chukeatirote E Kang JC Xu JC McKenzie EHC Jones EBG Hyde KD (2014) A molecular phylogenetic reappraisal of the Didymosphaeriaceae (= Montagnulaceae). Fungal Diversity 68: 69–104. 10.1007/s13225-014-0305-6

[B3] Ariyawansa HA Phukhamsakda C Thambugala KM Bulgakov TS Wanasinghe DN Perera RH Mapook A Camporesi E Kang JC Jones EBG Bahkali AH Jayasiri SC Hyde KD Liu ZY (2015) Revision and phylogeny of Leptosphaeriaceae. Fungal Diversity 74: 19–51. 10.1007/s13225-015-0349-2

[B4] Ariyawansa HA Hyde KD Liu JK Wu SP Liu ZY (2016a) Additions to Karst Fungi 1: *Botryosphaeriaminutispermatia* sp. nov., from Guizhou Province, China. Phytotaxa 275: 35–44. 10.11646/phytotaxa.275.1.4

[B5] Ariyawansa HA Hyde KD Thambugala KM Maharachchikumbura SSN Al-Sadi AM Liu ZY (2016b) Additions to Karst Fungi 2: *Alpestrisphaeriajonesii* from Guizhou Province, China. Phytotaxa 277: 255–265. 10.11646/phytotaxa.277.3.3

[B6] Avise JC Ball RMJ (1990) Principles of genealogical concordance in species concepts and biological taxonomy. Oxford surveys in evolutionary biology 7: 45–67.

[B7] Ban S Sakane T Nakagiri A (2015) Three new species of *Ophiocordyceps* and overview of anamorph types in the genus and the family Ophiocordycipitaceae Mycological progress 14(1): 1017. 10.1007/s11557-014-1017-8

[B8] Carbone I Kohn LM (1999) A method for designing primer sets for speciation studies in filamentous ascomycetes. Mycologia, 553–556. 10.2307/3761358

[B9] Chang JH Wang YZ (2009) The genera *Sporormia* and *Preussia* (Sporormiaceae, Pleosporales) in Taiwan. Nova Hedwigia 88(1/2): 245–254. 10.1127/0029-5035/2009/0088-0245

[B10] Chen Q Hou LW Duan WJ Crous PW Cai L (2017) Didymellaceae revisited. Studies in Mycology 87: 105–159. 10.1016/j.simyco.2017.06.002 28706324 PMC5498420

[B11] Dettman JR Jacobson DJ Turner E Pringle A Taylor JW (2003) Reproductive isolation and phylogenetic divergence in *Neurospora*: comparing methods of species recognition in a model eukaryote. Evolution 57: 2721–2741. 10.1111/j.0014-3820.2003.tb01515.x 14761052

[B12] Hennings PC (1900) Fungi japonica. Botanische Jahrbücher für Systematik, Pflanzengeschichte und Pflanzengeographie. Pflanzengeogr 28: 259–280

[B13] Hernández-Restrepo M Gené J Castañeda-Ruiz RF Mena-Portales J Crous PW Guarro J (2017) Phylogeny of saprobic microfungi from Southern Europe. Studies in Mycology 86: 53–97. 10.1016/j.simyco.2017.05.002 28626275 PMC5470572

[B14] Hyde KD Jones EBG Liu JK Ariyawansa H Boehm E Boonmee S Braun U Chomnunti P Crous PW Dai DQ Diederich P Dissanayake A Doilom M Doveri F Hongsanan S Jayawardena R Lawrey JD Li YM Liu YX Lücking R Monkai J Muggia L Nelsen MP Pang KL Phookamsak R Senanayake IC Shearer CA Suetrong S Tanaka K Thambugala KM Wijayawardene NN Wikee S Wu HX Zhang Y Aguirre-Hudson B Alias SA Aptroot A Bahkali AH Bezerra JL Bhat DJ Camporesi E Chukeatirote E Gueidan C Hawksworth DL Hirayama K Hoog SD Kang JC Knudsen K Li WJ Li XH Liu ZY Mapook A McKenzie EHC Miller AN Mortimer PE Phillips AJL Raja HA Scheuer C Schumm F Taylor JE Tian Q Tibpromma S Wanasinghe DN Wang Y Xu JC Yacharoen S Yan JY Zhang M (2013) Families of Dothideomycetes. Fungal Diversity 63: 1–313. 10.1007/s13225-013-0263-4

[B15] Hyde KD Nilsson RH Alias SA Ariyawansa HA Blair JE Cai L de Cock AWAM Dissanayake AJ Glockling SL Goonasekara ID Gorczak M Hahn M Jayawardena RS van Kan JAL Laurence MH Lévesque CA Li X Liu JK Maharachchikumbura SSN Manamgoda DS Martin FN McKenzie EHC McTaggart AR Mortimer PE Nair PVR Pawłowska J Rintoul TL Shivas RG Spies CFJ Summerell BA Taylor PWJ Terhem RB Udayanga D Vaghefi N Walther G Wilk M Wrzosek M Xu JC Yan JY Zhou N (2014) One stop shop: backbones trees for important phytopathogenic 5 genera: I (2014) Fungal Diversity 67(1): 21–125. 10.1007/s13225-014-0298-1

[B16] Jaklitsch WM Checa J Blanco MN Olariaga I Tello S Voglmayr H (2017) A preliminary account of the Cucurbitariaceae Studies in Mycology. 10.1016/j.simyco.2017.11.002 PMC5738211 29276320

[B17] Jaklitsch WM Voglmayr H (2016) Hidden diversity in *Thyridaria* and a new circumscription of the Thyridariaceae. Studies in Mycology 85: 35–64. 10.1016/j.simyco.2016.09.002 27872508 PMC5109270

[B18] Liu YJ Whelen S Hall BD (1999) Phylogenetic relationships among ascomycetes: evidence from an RNA polymerase II subunit. Molecular Biology and Evolution 16: 1799–1808. 10.1093/oxfordjournals.molbev.a026092 10605121

[B19] Liu YX Hyde KD Ariyawansa HA Li WJ Zhou DQ Yang YL Chen YM Liu ZY (2013) Shiraiaceae, new family of Pleosporales (Dothideomycetes, Ascomycota). Phytotaxa 103(1): 51–60. 10.11646/phytotaxa.103.1.4

[B20] Luangsa-ard J Tasanathai K Thanakitpipattana D Khonsanit A Stadler M (2018) Novel and interesting *Ophiocordyceps* spp. (Ophiocordycipitaceae, Hypocreales) with superficial perithecia from Thailand. Studies in Mycology 89: 125–142. 10.1016/j.simyco.2018.02.001 29910519 PMC6002337

[B21] Manamgoda DS Rossman AY Castlebury LA Crous PW Madrid H Chukeatirote E Hyde KD (2014) The genus *Bipolaris*. Studies in Mycology 79: 221–288. 10.1016/j.simyco.2014.10.002 25492990 PMC4255534

[B22] Miller MA Pfeiffer W Schwartz T (2010) Creating the CIPRES Science Gateway for inference of large phylogenetic trees. Proceedings of the Gateway Computing Environments Workshop (GCE), 14 Nov. 2010, New Orleans, LA, 1–8. 10.1109/GCE.2010.5676129

[B23] Morakotkarn D Kawasaki H Tanaka K Okane I Seki T (2008) Taxonomic characterization of Shiraia-like fungi isolated from bamboos in Japan. Mycoscience 49(4): 258–265. 10.1007/S10267-008-0419-3

[B24] Nylander J (2004) MrModeltest v2. Program distributed by the author, Evolutionary Biology Centre, Uppsala University, Uppsala, Sweden.

[B25] Page RD (2001) TreeView. Glasgow University, Glasgow, UK.

[B26] Rambaut A Drummond AJ (2007) Tracer v 1.4. http://beast.bio.ed.ac.uk/Tracer [Accessed 10 December 2017]

[B27] Rambaut A Drummond AJ (2008) FigTree: Tree figure drawing tool, version 1.2.2. http://tree.bio.ed.ac.uk/software/figtree/

[B28] Ronquist F Huelsenbeck JP (2003) MrBayes 3: Bayesian phylogenetic inference under mixed models. Bioinformatics 19(12): 1572–1574. 10.1093/bioinformatics/btg180 12912839

[B29] Schoch CL Seifert KA Huhndorf S Robert V Spouge JL Levesque CA Chen W Fungal Barcoding Consortium (2012) Nuclear ribosomal internal transcribed spacer (ITS) region as a universal DNA barcode marker for Fungi. Proceedings of the National Academy of Sciences 109: 6241–6246. 10.1073/pnas.1117018109 PMC3341068 22454494

[B30] Schoch CL Robbertse B Robert V Vu D Cardinali G Irinyi L Meyer W Nilsson RH Hughes K Miller AN Kirk PM Abarenkov K Aime MC Ariyawansa HA Bidartondo M Boekhout T Buyck B Cai Q Chen J Crespo A Crous PW Damm U Beer ZWD Dentinger BTM Divakar PK Dueñas M Feau N Fliegerova K García MA Ge ZW Griffith GW Groenewald JZ Groenewald M Grube M Gryzenhout M Gueidan C Guo L Hambleton S Hamelin R Hansen K Hofstetter V Hong SB Houbraken J Hyde KD Inderbitzin P Johnston PR Karunarathna SC Kõljalg U Kovács GM Kraichak E Krizsan K Kurtzman CP Larsson KH Leavitt S Letcher PM Liimatainen K Liu JK Lodge DJ Luangsaard JJ Lumbsch HT Maharachchikumbura SSN Manamgoda D Martín MP Minnis AM Moncalvo JM Mulè G Nakasone KK Niskanen T Olariaga I Papp T Petkovits T Pino-Bodas R Powell MJ Raja HA Redecker D Sarmiento-Ramirez JM Seifert KA Shrestha B Stenroos S Stielow B Suh SO Tanaka K Tedersoo L Telleria MT Udayanga D Untereiner WA Uribeondo JD Subbarao KV Vágvölgyi C Visagie C Voigt K Walker DM Weir BS Weiß M Wijayawardene NN Wingfield MJ Xu JP Yang ZL Zhang N Zhuang WY Federhen S (2014) Finding needles in haystacks: linking scientific names, reference specimens and molecular data for Fungi Database, 2014. 10.1093/database/bau061 PMC4075928 24980130

[B31] Stamatakis A (2014) RAxML version 8: a tool for phylogenetic analysis and post-analysis of large phylogenies. Bioinformatics 30: 1312–1313. 10.1093/bioinformatics/btu033 24451623 PMC3998144

[B32] Stielow B Bubner B Hensel G Munzenberger B Hoffmann P Klenk HP Göker M (2010) The neglected hypogeous fungus *Hydnotryabailii* Soehner (1959) is a widespread sister taxon of *Hydnotryatulasnei* (Berk.) Berk. and Broome (1846). Mycological Progress 9: 195–203. 10.1007/s11557-009-0625-1

[B33] Sung GH Sung JM Hywel-Jones NL Spatafora JW (2007) A multi-gene phylogeny of Clavicipitaceae (Ascomycota, Fungi): Identification of localized incongruence using a combinational bootstrap approach. Molecular Phylogenetics and Evolution 44: 1204–1223. 10.1016/j.ympev.2007.03.011 17555990

[B34] Sun JZ Dong CH Liu XZ Liu JK Hyde KD (2016) *Calcarisporiumcordycipiticola* sp. nov., an important fungal pathogen of *Cordycepsmilitaris*. Phytotaxa 268(2): 135–144. 10.11646/phytotaxa.268.2.4

[B35] Swofford D (2002) PAUP*. Phylogenetic analysis using parsimony (*and other methods). Version 4.0. Sinauer Associates. Sunderland, Massachusetts.

[B36] Tamura K Peterson D Peterson N Stecher G Nei M Kumar S (2011) MEGA5: molecular evolutionary genetics analysis using maximum likelihood, evolutionary distance, and maximum parsimony methods. Molecular Biology and Evolution 28: 2731–2739. 10.1093/molbev/msr121 21546353 PMC3203626

[B37] Tanaka K Hirayama K Yonezawa H Sato G Toriyabe A Kudo H Hashimoto A Matsumura M Harada Y Kurihara Y Shirouzu T Hosoya T (2015) Revision of the Massarineae (Pleosporales, Dothideomycetes). Studies in Mycology 82: 75–136. 10.1016/j.simyco.2015.10.002 26955201 PMC4774272

[B38] Taylor JW Jacobson DJ Kroken S Kasuga T Geiser DM Hibbett DS Fisher MC (2000) Phylogenetic species recognition and species concepts in fungi. Fungal Genetics and Biology 31: 21–32. 10.1006/fgbi.2000.1228 11118132

[B39] Tennakoon DS Phookamsak R Kuo CH Goh TK Jeewon R Hyde KD (2018) Morphological and phylogenetic evidence reveal *Fissuromataiwanense* sp. nov. (Aigialaceae, Pleosporales) from *Hedychiumcoronarium*. Phytotaxa 338(3): 265–275. 10.11646/phytotaxa.338.3.4

[B40] Thambugala KM Hyde KD Tanaka K Tian Q Wanasinghe DN Ariyawansa HA Jayasiri SC Boonmee S Camporesi E Hashimoto A Hiramaya K Schumacher RK Promputtha I Liu ZY (2015) Towards a natural classification and backbone tree for Lophiostomataceae, Floricolaceae, and Amorosiaceae fam. nov. Fungal Diversity 74(1): 199–266. 10.1007/s13225-015-0348-3

[B41] Tsai I Maharachchikumbura SSN Hyde KD Ariyawansa HA (2018) Molecular phylogeny, morphology and pathogenicity of *Pseudopestalotiopsis* species of Ixora. Mycological Progress. 10.1007/s11557-018-1404-7

[B42] Udayanga D Castlebury LA Rossman AY Chukeatirote E Hyde KD (2014) Insights into the genus *Diaporthe*: phylogenetic species delimitation in the *D.eres* species complex. Fungal Diversity 67(1): 203–229. 10.1007/s13225-014-0297-2

[B43] Uilenberg G Thiaucourt F Jongejan F (2004) On molecular taxonomy: what is in a name?. Experimental & applied acarology 32(4): 301–312. 10.1023/B:APPA.0000023235.23090.a7 15176735

[B44] Valenzuela-Lopez N Cano-Lira JF Guarro J Sutton D A Wiederhold N Crous PW Stchigel AM (2018) Coelomycetous Dothideomycetes with emphasis on the families Cucurbitariaceae and Didymellaceae. Studies in mycology 90: 1–69. 10.1016/j.simyco.2017.11.003 29255336 PMC5725287

[B45] Wanasinghe DN Hyde KD Crous PW Wijayawardene NN Jones EBG Bhat DJ Phillips AJL Groenewald JZ Dayarathne MC Phukhamsakda C Thambugala KM Bulgakov TS Camporesi E Gafforov YS Mortimer PE Karunarathna SC (2017) Phylogenetic revision of *Camarosporium* (Pleosporineae, Dothideomycetes) and allied genera. Studies in Mycology 87: 207–256. 10.1016/j.simyco.2017.08.001 28966419 PMC5607397

[B46] White TJ Bruns TD Lee S Taylor J (1990) Amplification and direct sequencing of fungal ribosomal RNA genes for phylogenetics. PCR protocols: a guide to methods and applications 18: 315–322. 10.1016/B978-0-12-372180-8.50042-1

[B47] Woudenberg JHC Groenewald JZ Binder M Crous PW (2013) *Alternaria* redefined. Studies in Mycology 75: 171–212. 10.3114/sim0015 24014900 PMC3713888

[B48] Yang JW Yeh YH Kirschner R (2016) A new endophytic species of *Neostagonospora* (Pleosporales) from the coastal grass *Spinifexlittoreus* in Taiwan. Botany 94(8): 593–598. 10.1139/cjb-2015-0246

[B49] Zhang Y Crous PW Schoch CL Hyde KD (2012) Pleosporales. Fungal Diversity 53: 1–221. 10.1007/s13225-011-0117-x 23097638 PMC3477819

[B50] Zhang Y Schoch CL Fournier J Crous PW Gruyter J Woundenberg JHC Hirayama K Tanaka K Pointing SB Spatafora JW Hyde KD (2009) Multi-locus phylogeny of Pleosporales: a taxonomic, ecological and evolutionary re–evaluation. Studies in Mycology 64: 85–102. 10.3114/sim.2009.64.04 20169024 PMC2816967

